# International medical graduates (IMGs) needs assessment study: comparison between current IMG trainees and program directors

**DOI:** 10.1186/1472-6920-8-42

**Published:** 2008-08-29

**Authors:** Rachelle Zulla, Mark Otto Baerlocher, Sarita Verma

**Affiliations:** 1 Diagnostic Radiology Residency Training Program, Faculty of Medicine, University of Toronto, Toronto, Ontario, Canada; 2 Postgraduate Medical Education, Faculty of Medicine, University of Toronto, Toronto, Ontario, Canada

## Abstract

**Background:**

International Medical Graduates (IMGs) training within the Canadian medical education system face unique difficulties. The purpose of this study was to explore the challenges IMGs encounter from the perspective of trainees and their Program Directors.

**Methods:**

Program Directors of residency programs and IMGs at the University of Toronto were anonymously surveyed and asked to rate (using a 5-point Likert scale; 1 = least important – 5 = most important) the extent to which specific issues were challenging to IMGs and whether an orientation program (in the form of a horizontal curriculum) should be implemented for incoming IMGs prior to starting their residency.

**Results:**

Among the IMGs surveyed, Knowledge of the Canadian Healthcare System received the highest mean score (3.93), followed by Knowledge of Pharmaceuticals and Hospital formularies (3.69), and Knowledge of the Hospital System (3.69). In contrast, Program Directors felt that Communication with Patients (4.40) was a main challenge faced by IMGs, followed by Communication with Team Members (4.33) and Basic Clinical Skills (4.28).

**Conclusion:**

IMGs and Program Directors differ in their perspectives as to what are considered challenges to foreign-trained physicians entering residency training. Both groups agree that an orientation program is necessary for incoming IMGs prior to starting their residency program.

## Background

Several studies note that International Medical Graduates (IMGs) experience additional problems relative to their Canadian counterparts such as loneliness, social isolation, concerns related to family members left behind in home countries, a decrease in social status with an accompanying diminishment of self-esteem, lack of financial resources and worries about visas/immigration issues [1,3]. Although some demographic commonalities exist among IMGs (such as generally being older, previously trained and/or practicing, and responsible for supporting a family), heterogeneity is also found due to variations in their life stages and perceptions of health and health care [4,5]. Furthermore, the level of medical training they have received prior to entering residency varies, which may prove challenging to supervisors. To address the difficulties IMGs face, previous studies find that exposure to an orientation program prior to entering postgraduate training is beneficial [6].

However, most orientation programs tend to concentrate on a specific skill, namely communication or cultural-sensitivity training. In a pilot study conducted at one Canadian medical school, IMGs participated in an 18-hour communication program, focusing on doctor-patient and doctor-colleague interaction during the first year of residency. Although the sample size was small, self-evaluations scores showed significant improvement after these sessions [7]. In another study, IMGs in one residency program were divided into two experimental groups to measure Emotional Resilience, Flexibility, Perceptual Acuity and Personal Autonomy. After cultural-sensitivity training, the experimental group (Group A) scored significantly higher in flexibility, emotional resilience and perceptual acuity than the control group. These IMGs were more self-confident, willing to learn from others, and able to correctly assess verbal and non-verbal cues [3].

In light of this, a needs assessment study was conducted at the University of Toronto to explore the extent that these specific issues were challenging for IMGs in a residency program from the perspective of Program Directors and foreign-trained physicians themselves.

## Methods

### Survey Instrument

Separate surveys were created for IMGs and Program Directors using iterative input from an expert panel of three faculty members. These individuals have worked with IMGs over the past four years with particular emphasis on mentoring, teaching and developing remediation plans.

Ten common issues were posed to both groups and covered the following three topics: Clinical Knowledge and Skills (3), Communication and Working Relations (4), and Macro Issues (3). These issues were identified by the expert panel as being the most pertinent and common across all subspecialties. For the purpose of this study, Macro Issues were defined as areas of concern affecting institutional, environmental or cultural experience and performance.

Currently, there is limited research exploring the impact of macro-level issues as challenges to the medical training of IMGs. Adapting to a new environment involves much more than learning how to communicate effectively. Essentially, the culture and principles, as well as the rules and procedures inherent in everyday operations of an organization, must be learned. Whether this translates to learning how to request a MRI or how to use software to track a patient's medical records, experiencing difficulty in adapting to a new environment may be mitigated by learning about these issues. Hence, under this premise, the survey included macro-level issues such as knowledge of the Canadian Healthcare System, knowledge of the hospital system (e.g. how to use pagers and computer software as well as adapting to hospital protocols) and hospital and pharmaceutical formularies.

Using a 5-point Likert scale, IMGs indicated to what extent each issue posed as a challenge to them during their residency. Program Directors were asked to what extent these issues were challenges to IMGs using the same scale. Further, both groups were asked whether an orientation program, in the form of a horizontal curriculum, was necessary for incoming IMGs. For this study, a horizontal curriculum is defined as a course of study with specific learning objectives for the purpose of obtaining a goal (i.e. providing a smoother transition of IMGs into residency programs at the University of Toronto).

### Recruitment

In the 2005 – 2006 academic year, a total of 118 IMGs were enrolled in all residency programs at the University of Toronto, as identified through the Postgraduate Medical Education registration database. IMGs were defined as individuals (regardless of citizenship status and source of funding) who obtained their medical degree outside of Canada at an accredited medical school, and training in a residency program recognized by the Royal College of Physicians and Surgeons of Canada and/or the College of Family Physicians of Canada. With the University of Toronto being the largest medical school in Canada offering almost all specialty and subspecialty residency training programs, 73 residency Program Directors were identified as potential participants.

Due to the number of IMGs and the difficulty in recruiting (i.e. multiple training sites), email invitations were sent out which included a link to complete the survey on-line. To increase the response rate, residents were given the option to include their email address for a chance to win an iPOD.

Email invitations were sent out to all Program Directors with the survey attached, as well as instructions on how to complete the on-line questionnaire. In the event that the former was chosen, Program Directors were given the option to fax or email completed surveys. To encourage the participation of Program Directors, these multiple accommodating methods were offered. Three follow-up emails were sent to IMGs and two follow-up emails were sent to Program Directors.

Participation for both groups was voluntary and subjects were given the option to withdraw at any time. Ethics approval was obtained by the University of Toronto Research Ethics Board for this study.

### Analysis

Frequencies for all eleven close-ended questions were calculated, as well as the mean rating for each of the 10 Likert-scale questions.

## Results

The response rate for IMGs was 74% (87 out of 118) and the response rate for Program Directors was 62% (45 out of 73). Mean scores for all ten topics and corresponding standard deviations are presented in Table 1.

**Table 1:** Mean scores of challenges faced by IMGs from the perspective of IMGs and their program directors

	**IMGs**	**PROGRAM DIRECTORS**
	**M (SD)**	**M (SD)**
**Knowledge and skills**		
Basic clinical skills	2.78 (1.195)	4.28 (1.007)
Specialty-specific clinical knowledge	3.28 (1.227)	3.37 (1.176)
Specialty-specific clinical skills	3.33 (1.198)	3.46 (1.031)
**Communication and working relationships**		
Communication with patients	3.49 (1.200)	4.40 (0.698)
Communication with team members	3.41 (1.177)	4.33 (0.644)
Working relations with other residents	3.05 (1.219)	3.80 (0.631)
Working relations with other healthcare students and allied healthcare professionals	3.13 (1.228)	4.00 (0.577)
**Macro issues**		
Knowledge of the Canadian Healthcare System	3.93 (1.097)	3.55 (0.852)
Knowledge of pharmaceuticals and Hospital Formularies	3.69 (0.980)	3.56 (0.959)
Knowledge of Hospital System	3.69 (1.060)	3.19 (0.764)

M = mean score

SD = standard deviation

Among the IMGs surveyed, Knowledge of the Canadian Health Care System received the highest mean score, followed by Knowledge of Pharmaceuticals and Hospital formularies (3.69), and Knowledge of Hospital Systems (3.69). The lowest mean rating scores were Basic Clinical Skills (2.78), Working Relations with Other Residents (3.05) and Working Relations with Other Healthcare Students and Allied Healthcare Professionals (3.13).

In contrast, the three highest mean scores among Program Directors were Communication with Patients (4.40), Communication with Team Members (4.33) and Basic Clinical Skills (4.28). The lowest mean scores were Knowledge of Hospital Systems (3.19), Specialty-specific Clinical Knowledge (3.37), and Specialty-specific Clinical Skills (3.46). Mean scores for Program Directors were higher relative to IMG trainees for all challenges presented in two categories: Knowledge and Skills and Communication and Working Relations. Mean scores for all Macro Issues were lower among Program Directors relative to the mean scores of IMGs.

Approximately three-quarters of all participants agreed that an orientation program specifically targeted for IMGs is needed. When broken down by group, 93% of all Program Directors surveyed believed that an orientation program was necessary. A smaller majority (63%) of IMGs believed such a program would be beneficial.

## Conclusions and discussion

Among the IMGs and Program Directors surveyed at our medical school, the majority agreed that an orientation program for all IMGs is required before starting a residency program. Such a program is currently provided for IMGs in the Faculty of Medicine and is reported to be insufficient (Childs and Herbert, Assessing IMG Performance at Ontario Medical Schools 2002–06 Final Report, December 2007). This finding is supported by previous work conducted in the United States. Mylonakis, Mega, and Schiffman [8] found that 37% of US Internal Medicine Program Directors agreed that a pre-residency training program should be mandatory prior to IMGs entering training. Other studies by Levey [9], Kidd and Zulman [10] and Kramer [1] found similar results.

Mean scores for Basic Clinical Skills differed between IMGs and Program Directors and may be related to the variability of training IMGs possess prior to entering the Canadian medical education system. With regards to Communication and Working Relations, mean scores were relatively higher among Program Directors, which may indicate they are more concerned with the communication skills and interprofessionalism of IMGs, in comparison to IMGs themselves. This is consistent with previous literature [11,7].

These findings are not exclusive to foreign-trained physicians. Yahes and Dunn [12] found that the biggest challenge experienced by foreign-trained nurses was a lack of communication skills. After completing two-hour sessions over the course of 12 weeks (dealing with group interaction, non-verbal cues, pronunciation and voice intonation) subjects reported a higher rate of job satisfaction and collegiality. Nursing administrators also noted a decrease in the number of complaints from physicians and staff with regards to communicating with these foreign-trained nurses.

All three Macro-level Issues (Knowledge of the Canadian Health Care System, Knowledge of Pharmaceutical and Hospital Formularies and Knowledge of Toronto Hospital Systems) received the highest mean scores among IMGs. System issues may prove especially challenging to IMGs who are required to learn the values of a new country's healthcare system and the culture of a hospital and its numerous administrative protocols, in addition to their medical curriculum. Program Directors may feel such issues are not challenges or may only view these as pertinent once the trainee begins his/her residency.

These concurrent surveys of IMG residents and their Program Directors at the University of Toronto illustrated that both cohorts feel that IMGs need better integration into their residency programs. Interestingly, the groups differed in the areas of concern with residents acknowledging their anxiety about a lack of knowledge and comfort in many macro areas, namely institutional and societal transition areas, whereas Program Directors were more concerned with areas of performance indicators in communication, collaboration and basic clinical skills. Each group responded based on their needs and anxieties, possibly indicating that they need to be educated about each other's perspectives.

IMGs are forming a major part of our training cohort, thus representing an increasing number of physicians entering into practice as a short-term solution to health human resource needs. A proliferation of IMG entries has not been accompanied by robust strategies to optimize their integration and bridge their transition into residency. In this study, IMGs have identified specific challenges as have their Program Directors. A comprehensive and integrated program is needed to facilitate the success of IMGs.

### Limitations

The project was designed as a needs assessment study to explore the extent to which specific issues were challenging to IMGs from the perspective of IMG trainees and Program Directors. As it stands, the survey instrument has only face validity.

Secondly, this study is exploratory in nature. As the survey only provided pre-selected issues, participants were restricted in ranking the choices presented. Follow-up studies using focus groups with IMGs may help to expand on their experiences or uncover specifics that cannot be captured using close-ended questions. The same qualitative methods should be used to explore the perceptions of Program Directors, not only for contrast, but also to identify other issues that may not be shared by the IMG cohort.

Conclusions of this study are specific to the perceptions of IMGs and Program Directors of medical residency programs at the Faculty of Medicine at the University of Toronto. It cannot be concluded that IMGs and Program Directors at other Canadian medical schools or elsewhere perceive these issues as challenges, or to the same extent as those who participated in this study.

## Competing interests

Funds have been received from the Ontario Ministry of Health by the Postgraduate Medical Education Office to design an Orientation Program for incoming IMGs. Results from this study were strictly used as one component to assist the Director of Education and Research in designing the program. No formal report of this study has been disseminated to this funder or other sponsors.

The Postgraduate Medical Education Office is financing this manuscript, including the article-processing fee. This office employs both Dr. Sarita Verma, Vice-Dean, and Rachelle Zulla, Research and Data Analyst. Dr. Baerlocher is currently a resident in the Department of Medical Imaging. None of the contributing authors hold stocks or shares in this organization and no patents related to the content of the manuscript have been submitted. All authors declare that they have no competing interests.

## Authors' contributions

SV is responsible for the conception and design of this study, as well as critically revising the final draft. The acquisition of data, analysis and interpretation of data, as well as writing and editing the final version, was done by RZ. MOB contributed by interpreting the data and contributing to writing the final version of this paper. All authors have read and approved this manuscript.

## Pre-publication history

The pre-publication history for this paper can be accessed here:


